# X-linked inhibitor of apoptosis protein is a prognostic marker for a favorable outcome in three identified subsets in resectable adenocarcinoma of the pancreas

**DOI:** 10.1007/s00432-022-04476-2

**Published:** 2022-12-06

**Authors:** Karl Knipper, Su Ir Lyu, Heike Goebel, Alexander I. Damanakis, Yue Zhao, Christiane J. Bruns, Thomas Schmidt, Hamid Kashkar, Alexander Quaas, Lars M. Schiffmann, Felix C. Popp, the PANCALYZE Study Group

**Affiliations:** 1 Faculty of Medicine, Department of General, Visceral and Cancer Surgery, University Hospital of Cologne, University of Cologne, Cologne, Germany; 2 Faculty of Medicine, Institute of Pathology, University Hospital of Cologne, University of Cologne, Cologne, Germany; 3 Faculty of Medicine, Institute for Molecular Immunology, University Hospital of Cologne, University of Cologne, Cologne, Germany

**Keywords:** Pancreatic ductal adenocarcinoma, X-linked inhibitor of apoptosis protein, Inflammatory microenvironment, Personalized medicine

## Abstract

**Purpose:**

Pancreatic ductal adenocarcinoma (PDAC) is currently one of the leading causes of cancer death worldwide. Therefore, building further subgroups as well as enabling individual patient therapy and diagnostics are needed. X-linked inhibitor of apoptosis protein (XIAP) is known to modulate apoptotic and inflammatory pathways. Its expression was found to correlate with patients’ survival in other tumor entities. This study aims to examine the role of XIAP in patients with PDAC in relation to the inflammatory microenvironment.

**Methods:**

The PANCALYZE multicenter study group included 257 patients with PDAC. Paraffin-embedded tumor samples were stained immunohistochemically for CD3, CD20, CD38, CD56, CD66b, CD117, and CD163 and XIAP. These stainings were further analyzed digitally with QuPath and survival analyses were done.

**Results:**

XIAP-positive patients with T-cell, respectively, neutrophil enriched tumors survived significantly longer compared to XIAP-negative patients (CD3: 37.6 vs. 24.6 months, *p* = 0.028; CD66b: 34.1 vs. 14.9 months, *p* = 0.027). Additionally, XIAP-positive patients showed better survival in the lymph node-negative population (48.4 vs. 24.2 months, *p* = 0.019). Regarding the total population, our findings did not show a correlation between XIAP expression and survival. In multivariate cox regression analyzes XIAP proves to be an independent factor for better survival in the identified subgroups (CD3: *p* = 0.043; CD66b: *p* = 0.012, N0: *p* = 0.040).

**Conclusion:**

We found XIAP-positive subgroups with significantly better survival in patients with PDAC in T-cell-rich, neutrophil-rich, or lymph node-negative cohorts. This could lead to further individualized cancer treatment with less aggressive therapy protocols for XIAP-positive tumors or more intensive follow-up for XIAP-negative tumors.

**Supplementary Information:**

The online version contains supplementary material available at 10.1007/s00432-022-04476-2.

## Background

Pancreatic cancer is estimated to be the seventh most common cause of death worldwide (Ferlay et al. 2015). Pancreatic ductal adenocarcinoma (PDAC) makes up more than 90% of pancreatic cancer cases, with a 5-year survival rate of less than 4%. Due to a lack of specific symptoms and rapid tumor progression, only 10% of the patients are operable at the time of diagnosis (Hidalgo et al. 2015). To improve the overall outcome of resectable PDAC-patients establishing approachable survival predictors with a subsequently expanded therapeutic approach as well as possibly stricter post-therapeutic surveillance are needed.

A member of the apoptosis inhibitor family (IAP), the X-linked inhibitor of apoptosis protein (XIAP) is known for its anti-apoptotic activity and is expressed in numerous human tumors (Tamm et al. 2000; Kashkar 2010). Increased XIAP mRNA levels in pancreatic cancers were shown to have a tendency for reduced patient survival and contribute to the gemcitabine chemoresistance (Shrikhande et al. 2006). Like many other cell death regulators, IAPs also modulate the inflammatory signaling pathways (Vucic 2018). XIAP also induces proinflammatory signaling by involving receptor-interacting serine/threonine kinase 2 (RIPK2) and production of interleukin 8 (IL-8) which leads to neutrophil recruitment in cancer and during infection (Krieg et al. 2009; Andree et al. 2014; Daoud et al. 2022). Inactivating *XIAP* gene mutations are associated with inflammatory diseases like very early-onset inflammatory bowel disease and X-linked lymphoproliferative syndrome type-2 (Pedersen et al. 2014; Damgaard et al. 2013). This highlights not only the effects on apoptosis but also the inflammatory cell response regulative function of XIAP, turning it into an interesting research target considering inflammatory tumor microenvironment properties. This study aims to identify the relevance of XIAP expression as a predictor of overall survival in a large collection of operable pancreatic ductal adenocarcinoma and its correlation with the inflammatory microenvironment.

## Methods

### Patients and tumor samples

Formalin-fixed and paraffin-embedded samples of 257 patients with PDAC from primary surgical resection or after neoadjuvant therapy between 2014 and 2020 were analyzed. The patients were recruited by the PANCALYZE study group. Written informed consent was obtained from every patient. The study was approved by the local ethics committees and was conducted in accordance with the declaration of Helsinki. Three patients were excluded since no tumor was detected in the tissue sample, which was transferred by the study center. Surgeries were performed according to the German S3 guidelines for PDAC (Onkologie and (Deutsche Krebsgesellschaft DK, AWMF) 2021). TNM stage was interpreted following the 8^th^ edition of the Union for International Cancer Control.

For the tissue microarrays (TMA) construction a semi-automated precision instrument was used to punch tissue cylinders of 1.2 mm (two for each tumor sample), which were transferred into recipient paraffin blocks. For further immunohistochemistry 4 µm thick slices were transferred to an adhesive-coated slide system (Instrumedics Inc., Hackensack, NJ) (Simon 2010).

### Immunohistochemistry (IHC) and analysis

Using polyclonal rabbit XIAP-antibody as well as CD3, CD20, CD38, CD56, CD66b, CD117, and CD163 for inflammatory cell profile on Leica Bond-MAX automated system (Leica Biosystems, Germany) immunohistochemical stainings were performed according to the manufacturer's protocol (Supp. Table 1). The further evaluation of immunohistochemical expression cores for XIAP was performed by an experienced pathologist (H. Goebel). XIAP staining was interpreted as either negative or positive. Stained slides were scanned and digitalized with the NanoZoomer S360 (Hamamatsu, Japan). The immunohistochemical expression cores for the inflammatory cell profile were analyzed digitally via QuPath v0.3.2 (Bankhead et al. 2017). Here, we analyzed the entire stained samples. The mean of both tumor samples was assessed for each patient. To form further subgroups, the total population was divided in low and high expressing groups assessed by immunohistochemical staining (CD3: low < 110 cells/sample and high ≥ 110 cells/sample, CD 38: low < 15 cells/sample and high ≥ 15 cells/sample, CD66b: low < 158 cells/sample and high ≥ 158 cells/sample).

### Statistical analysis

Prospective clinical data gathering was performed according to a standardized protocol (Popp et al. 2017).

Data were analyzed retrospectively with IBM SPSS Statistics (Version 28.0.1.1). The Chi-square test was used to compare qualitative values. Overall survival was defined from the date of surgery until patients’ death or loss of follow-up. Survival curves were analyzed with the Kaplan–Meier method and log-rank test. To further evaluate interdependence between the clinicopathologic values and survival, multivariate Cox regression was used. Results of multivariate Cox regression are shown as forest plots. P-Values below 0.05 were considered statistically significant.

## Results

**Fig. 1:**
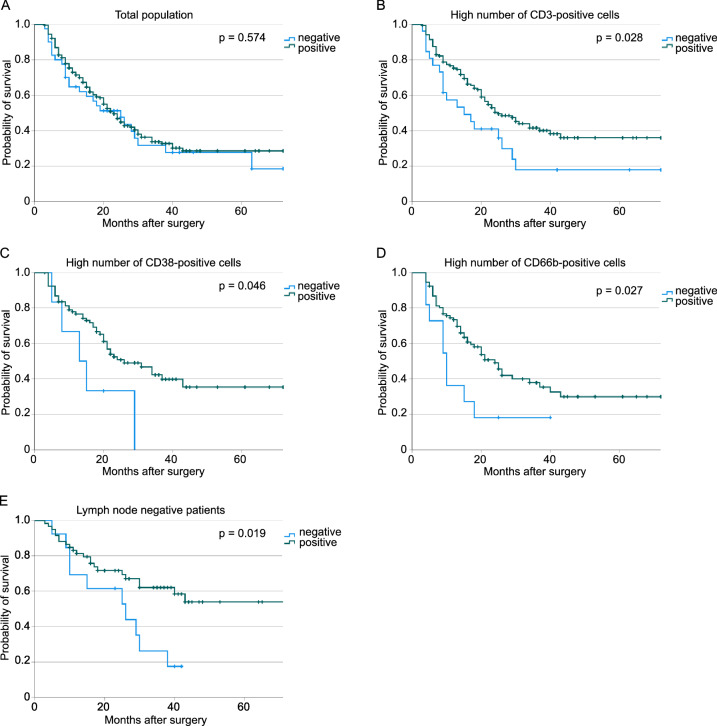
Kaplan–Meier curves for the overall survival comparing patients with XIAP-negative and -positive tissue: **a** total population (*n *(negative) = 40, *n*(positive) = 214, *p* = 0.574), **b** high number of CD 3-positive cells (n (negative) = 26, n (positive) = 152, *p* = 0.028), **c** high number of CD 38-positive cells (*n *(negative) = 6, *n *(positive) = 92, *p* = 0.046), **d** high number of CD 66b-positive cells (*n *(negative) = 11, *n *(positive) = 91, *p* = 0.027) and **e** lymph node negative patients (*n *(negative) = 13, *n *(positive) = 59, *p* = 0.019)

We included 254 patients in the final analysis (Table 1). 118 (46.5%) were male, 136 (53.5%) were female. The median age was 71 years (range: 42—97 years). The median follow-up was 18 months (range: 3—73 months). 211 (83.1%) patients suffered from an operable adenocarcinoma located in the pancreatic head, 30 (11.8%) in the corpus, and 3 (5.1%) in the pancreatic cauda. Before surgical resection 2% (*n* = 5) were treated with radio-chemotherapy. Immunohistochemical staining for XIAP was conducted in all patients. 15.7% of the samples were considered negative and 84.3% were positive. No significant differences in clinicopathologic values between these two groups were found (Table 1). Furthermore, the overall survival of these two groups did not differ (XIAP negative: OS: 30.60 ± 1.46 months, XIAP positive: OS: 33.41 ± 2.11 months, *p* = 0.574, Fig. 1a).

**Table 1:** Patients’ characteristics of the total population and the following subgroups: XIAP-negative, XIAP-positive, lymph node-negative, high CD3 infiltration, high CD38 infiltration, and high CD66b infiltration

Characteristic	Total	XIAP negative	XIAP positive	*P*-value	pN0	CD3 high	CD38 high	CD66b high
	*n* (%)	*n* (%)	*n* (%)		*n* (%)	*n* (%)	*n* (%)	*n* (%)
No. of patients	254 (100)	40 (100)	214 (100)		72 (100)	178 (100)	98 (100)	102 (100)
Sex				0.585				
Male	118 (46.5)	17 (42.5)	101 (47.2)		29 (40.3)	77 (43.3)	46 (46.9)	43 (42.2)
Female	136 (53.5)	23 (57.5)	113 (52.8)		43 (59.7)	101 (56.7)	51 (53.1)	59 (57.8)
Age				0.870				
< 65	79 (31.1))	12 (30.0)	67 (31.3)		14 (19.4)	55 (30.9)	36 (36.7)	21 (20.6)
≥ 65	175 (68.9)	28 (70.0)	147 (68.7)		58 (80.6)	123 (69.1)	62 (63.3)	81 (79.4)
Median overall survival (months)	18	19	18		26	18	20	16
(range)	(3–73)	(3–72)	(3–73)		(3–73)	(3–73)	(3–72)	(4–73)
Neoadjuvant therapy				–				
No	249 (98.0)	37 (92.5)	212 (99.1)		69 (95.8)	173 (97.2)	96 (98.0)	102 (100)
Chemotherapy	3 (1.2)	2 (5.0)	1 (0.5)		2 (2.8)	3 (1.7)	2 (2.0)	0 (0.0)
Radiochemotherapy	2 (0.8)	1 (2.5)	1 (0.5)		1 (1.4)	2 (1.1)	0 (0.0)	0 (0.0)
Localization				0.173				
Head	211 (83.1)	31 (77.5)	180 (84.1)		57 (79.2)	148 (83.1)	82 (83.7)	87 (85.3)
Corpus	30 (11.8)	8 (20.0)	22 (10.3)		14 (19.4)	23 (12.9)	12 (12.2)	11 (10.8)
Cauda	13 (5.1)	1 (2.5)	12 (5.6)		1 (1.4)	7 (3.9)	4 (4.1)	4 (3.9)
pT				0.322				
1	13 (5.1)	3 (7.5)	10 (4.7)		7 (9.7)	12 (6.7)	4 (4.1)	5 (4.9)
2	100 (39.4)	12 (30.0)	88 (41.1)		33 (45.8)	71 (39.9)	49 (50.0)	46 (45.1)
3	135 (53.1)	25 (62.5)	110 (51.4)		31 (43.1)	90 (50.6)	45 (45.9)	47 (46.1)
4	6 (2.4)	0 (0.0)	6 (2.8)		1 (1.4)	5 (2.8)	0 (0.0)	4 (3.9)
pN				0.703				
0	72 (28.3)	13 (32.5)	59 (27.6)		72 (100)	51 (28.7)	27 (27.6)	31 (30.4)
1	90 (35.4)	12 (30.0)	78 (36.4)		0 (0.0)	67 (37.6)	34 (34.7)	39 (38.2)
2	92 (36.2)	15 (37.5)	77 (36.0)		0 (0.0)	60 (33.7)	37 (37.8)	32 (31.4)
R				0.910				
0	158 (62.2)	25 (62.5)	133 (62.1)		55 (76.4)	112 (62.9)	58 (59.2)	58 (56.9)
1	95 (37.4)	15 (37.5)	80 (37.4)		17 (23.6)	65 (36.5)	40 (40.8)	44 (43.1)
2	1 (0.4)	0 (0.0)	1 (0.5)		0 (0.0)	1 (0.6)	0 (0.0)	0 (0.0)
Pn				0.493				
0	43 (16.9)	8 (20.0)	35 (16.4)		23 (31.9)	33 (18.5)	15 (15.3)	25 (24.5)
1	187 (73.6)	27 (67.5)	160 (74.8)		41 (56.9)	126 (70.8)	78 (79.6)	64 (62.7)
Unknown	24 (9.4)	5 (12.5)	19 (8.9)		8 (11.1)	19 (10.7)	5 (5.1)	12 (12.7)
L				0.147				
0	82 (32.3)	16 (40.0)	66 (30.8)		44 (61.1)	59 (33.1)	29 (29.6)	38 (37.3)
1	160 (63.0)	20 (50.0)	140 (65.4)		25 (34.7)	110 (61.8)	64 (65.3)	63 (61.8)
Unknown	12 (4.7)	4 (10.0)	8 (3.8)		3 (4.2)	9 (5.1)	5 (5.1)	1 (0.9)
V				0.468				
0	136 (53.5)	23 (57.5)	113 (52.8)		53 (73.6)	103 (57.9)	49 (50.0)	60 (58.8)
1	83 (32.7)	11 (27.5)	72 (33.6)		12 (16.7)	47 (26.4)	32 (32.7)	32 (31.4)
Unknown	35 (13.8)	6 (15.0)	29 (13.6)		7 (9.7)	28 (15.7)	17 (17.3)	10 (9.8)

Since it is known that XIAPs’ influence on patients’ outcomes varies with the different inflammatory microenvironments, we assessed infiltrating immune cells in the tumor tissue. For further analysis, the population was divided into additional subgroups based on the amounts of inflammatory cells in the tumor microenvironment separated into low and high numbers of infiltrating cells per sample. Representative images of the immunohistochemical stainings are shown in supplement Fig. 1. Each subgroup was then analyzed for the influence of XIAP on overall survival. Overall survival was favorable for patients with XIAP expression in subgroups with high numbers of T-lymphocytes, plasma cells or polymorph neutrophils (CD3: *p* = 0.028, CD38: *p* = 0.046, CD66b: *p* = 0.027, Fig. 1b–d). Additional multivariate Cox regression analyzes were performed to assess confounders and effect-modifiers, especially TNM-stage. XIAP expression proofs to be an independent protective factor for overall survival in subgroups of high infiltrating T-lymphocytes and neutrophils but not in the subgroup of plasma cells-high (CD3: HR: 0.568, CI: 0.328—0.982, *p* = 0.043, Table 2; CD38: HR: 0.366, CI 0.096—1.397, *p* = 0.141, Supp. Table 2; CD66b: HR: 0.352, CI 0.155—0.798, *p* = 0.012; Table 3). The N-status is an independent risk factor for worse survival in subgroups of high infiltrating T-lymphocytes and plasma cells (Table 2, Supp. Table 2).

**Table 2:**
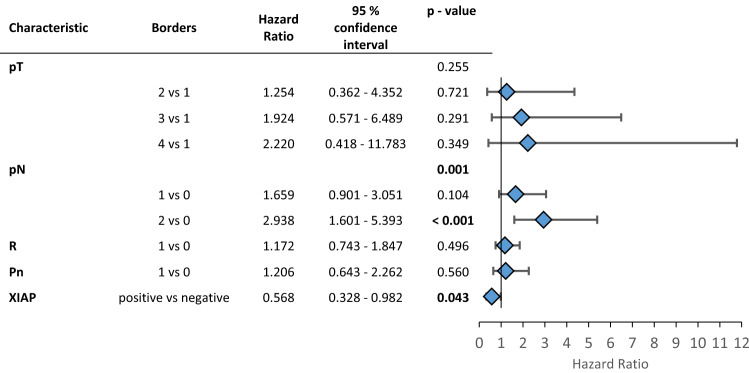
Multivariate cox regression for the patient subgroup with a high CD3 infiltration (T-cell-rich)

*P*-values below 0.05 are marked in bold

**Table 3:**
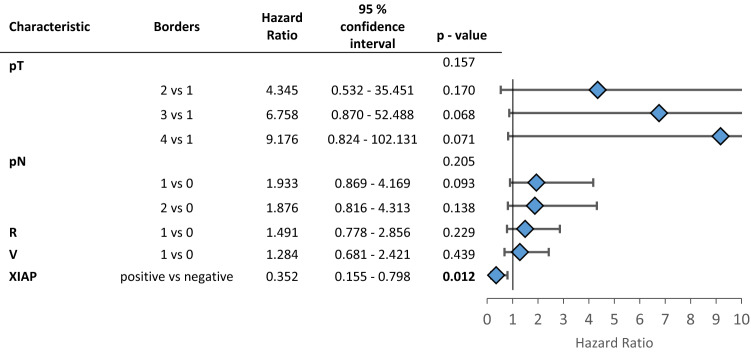
Multivariate cox regression for the patient subgroup with a high CD66b infiltration (neutrophil-rich)

*P*-values below 0.05 are marked in bold

Additionally, we assessed the role of XIAP in patients depending on their N-status. Indeed, XIAP expressing patients showed an improved overall survival in the subgroup of node-negative patients (XIAP negative: 24.17 ± 3.57 months, XIAP positive: 48.35 ± 4.06 months, *p* = 0.019, Fig. 1e). The multivariate Cox regression analysis showed that XIAP acts independently of other clinicopathologic factors for patients’ overall survival in lymph node-negative patients (HR: 0.423, CI 0.186—0.961, *p* = 0.040, Table 4). Additionally, blood vessel invasion is an independent marker for worse survival in this subgroup (Table 4).

**Table 4:**
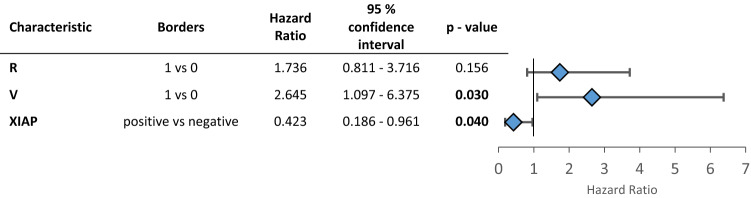
Multivariate cox regression for the patient subgroup without lymph node metastases

*P*-values below 0.05 are marked in bold

In summary, our data show that XIAP has no impact on survival when analyzed for the whole study population. However, when assessed in subgroups of lymph node-negative patients or patients with high infiltrating cell numbers of T-lymphocytes or granulocytes XIAP is an independent protective factor for patients’ outcomes.

## Discussion

In the current study, we analyzed XIAP expression in a cohort of 254 patients with operable pancreatic ductal adenocarcinoma. Our results showed no significant difference in overall survival depending on XIAP expression levels.

Considering the fact that XIAP does not only act as an anti-apoptotic protein but also shows immunomodulatory features, for example by inducing NF-κB (Lu et al. 2007), we divided our patient collective into two groups, depending on the tumors’ immune cell infiltration. Here, we saw that a positive XIAP expression in high CD3 (T-cell-rich) and CD66b (neutrophil-rich) infiltrated PDACs was significantly connected with longer overall survival and is even an independent protective factor. Similar to our observation, increased XIAP expression was a predictor of a positive outcome in other tumor types, for example, non-small-cell-lung cancer (NSCLS) and lower probability of tumor recurrence in prostate cancer (Ferreira et al. 2001; Seligson et al. 2007). High infiltration of CD3 itself has been shown to correlate with better overall and progression-free survival in PDAC (Miksch et al. 2019). Similar results were already observed with nuclear survivin-positive stainings in pancreatic adenocarcinoma (Tonini et al. 2005). Survivin is another important IAP family member. Its increased expression in PDAC could also be demonstrated to be associated with a longer overall survival (Tonini et al. 2005). Our results contradict the findings of Shrikhande et al. (Shrikhande et al. 2006) where overall survival was shorter in patients with higher tumor cell XIAP expression (*n* = 43). The discrepancy in the results could be due to evaluation methods (RT-PCR vs. IHC quantification) as well as the cohort size difference (*n* = 43 vs. *n* = 254) as different unbalanced subgroups in small collectives could bias the overall result.

The finding that the XIAP overexpression is associated with a favorable outcome is contradictory to findings from us and others in different tumor entities, such as esophageal cancer (Schiffmann et al. 2019) and melanoma (Daoud et al. 2022). In these publications, the role of XIAP in cancer progression was intimately linked to the microenvironment. Since PDAC inflammatory microenvironment differs from other tumor entities, it is possible that XIAP’s immunosuppressive impact shows the described duality on individual tumors. (Pushalkar et al. 2018). It is reasonable to hypothesize that based on mechanistic studies to the role of XIAP in cancer and non-malignant conditions (Daoud et al. 2022; Goncharov et al. 2018; Kashkar et al. 2006) increased tumor XIAP level trigger the recruitment of myeloid derived cells to the tumor which modulates the anti-tumor immune response. The hypothesis that this XIAP stimulated immune cell infiltrate is anti-tumoral for patients’ outcome in PDAC but detrimental in other entities and the role of XIAP in lymph node metastasis should be evaluated in further sophisticated mechanistic studies in animal as well as in vitro models.

Since neoadjuvant therapy has emerged as the standard of care for patients with locally advanced or borderline resectable PDAC (Petrelli et al. 2015; Jang et al. 2018), further predictors for therapy response are needed. Theoretically, XIAP expression could be measured in the, according to guidelines needed, biopsy before neoadjuvant therapy. Furthermore, XIAP expression has been correlated, among others, with cisplatin resistance in head and neck cancer (Yang et al. 2012). However, to transfer these findings into treatment of PDAC more mechanistic studies are needed.

As a clinical consequence of our work more frequent follow-up exams could be suggested for XIAP-negative PDAC patients with the above-described inflammatory cell infiltration status. This should be subject to further translational and clinical studies.

## Conclusions

Our data indicate XIAP as an independent predictor for overall survival in PDAC patients with high immune cell infiltration (particularly CD3 and CD66b) and highlights the importance of PDAC subtypes classification. This could help with additional mechanistic studies to form further subgroups for treatment and diagnostic algorithms.

## Supplementary Information

Below is the link to the electronic supplementary material.

Supplementary Figure 1 Representative pictures of immunohistochemical stainings with (a) XIAP (left: negative, right: positive), (b) CD3 (left: low, right: high), (c) CD38 (left: low, right: high), and (d) CD66b (left: low, right: high). Scale bar: 50 µm. Table 1 Detailed antibody information. A: appendix vermiformis, T: tonsilla palatina Table 2 Multivariate cox regression for the patient subgroup with a high CD38 infiltration (plasma-cell rich). *P*-values below 0.05 are marked in bold (DOCX 732 KB)

## Data Availability

The datasets generated and analyzed during the current study are available from the corresponding author on reasonable request.
