# Supplementary material for: X-linked inhibitor of apoptosis protein is a prognostic marker for a favorable outcome in three identified subsets in resectable adenocarcinoma of the pancreas
Source: J Cancer Res Clin Oncol. 2022 Dec 6;149(9):5531–8. doi: 10.1007/s00432-022-04476-2 (PMC10356682; doi:10.1007/s00432-022-04476-2)
Supplement: Supplementary file 1 — Supplementary Figure 1 Representative pictures of immunohistochemical stainings with (a) XIAP (left: negative, right: positive), (b) CD3 (left: low, right: high), (c) CD38 (left: low, right: high), and (d) CD66b (left: low, right: high). Scale bar: 50 µm. Table 1 Detailed antibody information. A: appendix vermiformis, T: tonsilla palatina Table 2 Multivariate cox regression for the patient subgroup with a high CD38 infiltration (plasma-cell rich). P-values below 0.05 are marked in bold (DOCX 732 KB) [file 432_2022_4476_MOESM1_ESM.docx]

**7. Supplement Information**

**Supp. Figure 1** Representative pictures of immunohistochemical stainings with (a) XIAP (left: negative, right: positive), (b) CD3 (left: low, right: high), (c) CD38 (left: low, right: high), and (d) CD66b (left: low, right: high). Scale bar: 50 µm.

**
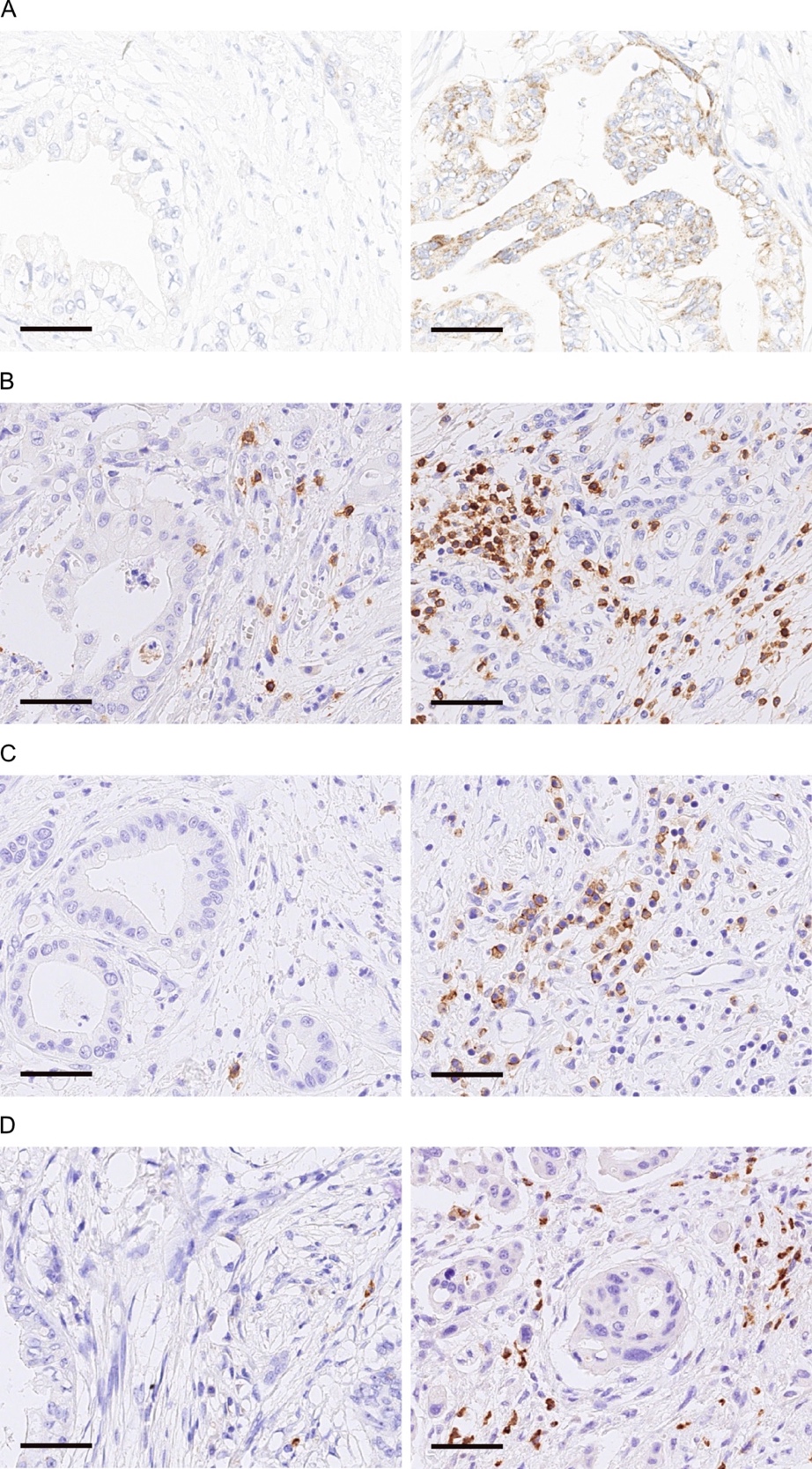
**

**Supp. Table 1** Detailed antibody information. A: appendix vermiformis, T: tonsilla palatina

| **Antibody** | **Manufacturer** | **Clone** | **Dilution** | **Pretreatment** | **Control** | **Order number** |
| --- | --- | --- | --- | --- | --- | --- |
| CD3 | Thermo | SP7, rabbit | 1:50 | Citrat | A/T | RM-9107-S |
| CD20 | Dako | L26, mouse | 1:1250 | Citrat | A/T | M0755 |
| CD38 | Novocastra | SPC32, mouse | 1:800 | Citrat | A/T | NCL-L-CD38-29 |
| CD56 | Thermo | 123C3 | 1:500 | EDTA | A | MA5-16445 |
| CD66b | Novusbio | G10F5 | 1:200 | EDTA | A/T | NB100-77808 |
| CD117 | Biocare Medical | EP10, rabbit | 1:50 | Citrat | sarcoma-TMA | CME296C |
| CD163 | Cellmarque | MRQ-26B-B4, mouse | 1:100 | EDTA | A/T | 163M-16 |
| XIAP | abcam | Polyclonal, rabbit | 1:1000 | Citrat |  | Ab21278 |

**Supp. Table 2** Multivariate cox regression for the patient subgroup with a high CD38 infiltration (plasma-cell rich). P-values below 0.05 are marked in bold.

| **Characteristic** | **Borders** | **Hazard Ratio** | **95 % confidence interval** | 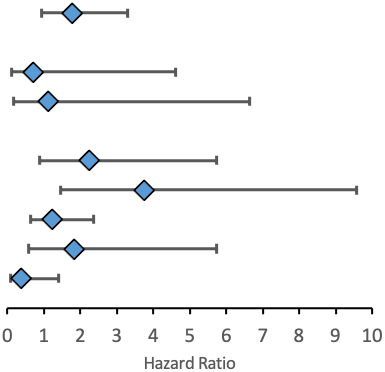 **p - value** |  |  |  |  |  |  |
| --- | --- | --- | --- | --- | --- | --- | --- | --- | --- | --- |
| **Sex** | female vs male | 1.763 | 0.943- 3.296 | 0.076 |  |  |  |  |  |  |
| **pT** |  |  |  | 0.366 |  |  |  |  |  |  |
|  | 2 vs 1 | 0.713 | 0.110 - 4.617 | 0.722 |  |  |  |  |  |  |
|  | 3 vs 1 | 1.118 | 0.164 - 7.640 | 0.909 |  |  |  |  |  |  |
| **pN** |  |  |  | **0.020** |  |  |  |  |  |  |
|  | 1 vs 0 | 2.251 | 0.884 - 5.729 | 0.089 |  |  |  |  |  |  |
|  | 2 vs 0 | 3.742 | 1.465 - 9.560 | **0.006** |  |  |  |  |  |  |
| **R** | 1 vs 0 | 1.240 | 0.648 - 2.372 | 0.515 |  |  |  |  |  |  |
| **Pn** | 1 vs 0 | 1.835 | 0.587 - 5.734 | 0.296 |  |  |  |  |  |  |
| **XIAP** | positive vs negative | 0.366 | 0.096 - 1.397 | 0.141 |  |  |  |  |  |  |
